# Effect of STW 5‐II (Iberogast‐N) on Tolerance to Gastric Gas in Patients With Functional Dyspepsia. The IBO‐2 Study

**DOI:** 10.1111/nmo.70123

**Published:** 2025-07-20

**Authors:** Ariadna Aguilar, Luis Alcala‐Gonzalez, Claudia Barber, Javier Santos, Beatriz Lobo, Carolina Malagelada, Jordi Serra

**Affiliations:** ^1^ Digestive System Research Unit University Hospital Vall d'Hebrón Barcelona Spain; ^2^ Autonomous University of Barcelona Barcelona Spain; ^3^ Centro de Investigación Biomédica en Red de Enfermedades Hepáticas y Digestivas (CIBERehd) Madrid Spain

**Keywords:** bloating, functional dyspepsia, gastric gas, STW 5‐II

## Abstract

**Background:**

STW 5‐II has been shown to improve numerous symptoms in functional dyspepsia.

**Aim:**

To determine if the herbal medicinal product STW 5‐II may improve gastric gas transit and tolerance in patients with functional dyspepsia.

**Methods:**

In a parallel, randomized, double‐blinded, placebo‐controlled study, a gas challenge test was performed in 32 patients with functional dyspepsia and bloating after 2 weeks of treatment with (a) STW 5‐II and (b) placebo. The challenge test consisted of continuous infusion of gas into the stomach (100 mL/min for 15 min) with simultaneous nutrient perfusion (315 kcal for 15 min, 210 mL final volume). Gas evacuation, symptom perception, and abdominal distension were continuously registered for 90 min.

**Results:**

Gastric gas infusion was followed by a progressive start of gas evacuation from the rectum that was significantly accelerated when patients were on STW 5‐II (319 ± 81 mL after 20 min infusion start), compared to placebo (80 ± 39 mL; *p* = 0.015), but this difference declined during the following 90 min of gas evacuation (final gas retention 470 ± 160 and 662 ± 179, STW 5‐II and placebo, respectively, *p* = 0.431). Gas infusion was associated with a significant rise and posterior progressive decline in abdominal symptom perception (mainly bloating) that was significantly lower in patients treated with STW 5‐II (mean score increment 0.8 ± 0.4) than in patients treated with placebo (score increment 2.1 ± 0.4; *p* = 0.036). There were no significant differences in abdominal distension between groups.

**Conclusion:**

STW 5‐II improves tolerance to gastric gas in patients with functional dyspepsia and may be beneficial for the treatment of gas‐related abdominal symptoms like bloating.

**Trial Registration:**

EudraCT number: 2019‐003976‐38

## Graphical abstract

In a parallel, placebo‐controlled study with direct gas infusion into the stomach, STW 5‐II reduced the perception of abdominal symptoms in patients with dyspepsia complaining of bloating when compared to placebo, with a minor effect on gas transit and evacuation, and no effect on visible abdominal distension.

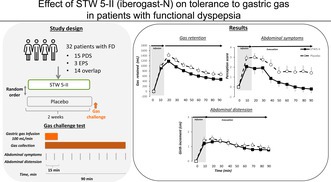

**Abbreviations**
FD: functional dyspepsia
FD‐EPS: functional dyspepsia–epigastric pain syndrome
FD‐PDS: functional dyspepsia–postprandial distress syndrome
SGB: supra‐gastric belching

**Summary**

- STW 5‐II is an herbal medicinal product that has been shown to improve numerous symptoms in functional dyspepsia. The effects of STW 5‐II on gastric gas transit and tolerance in functional dyspepsia have not been previously investigated.
- Using a parallel, placebo‐controlled study design with direct gas infusion into the stomach, STW 5‐II accelerated transit and rectal evacuation of gastric gas and reduced perception of symptoms like bloating when compared to placebo with no effect on visible abdominal distension.
- STW 5‐II improves tolerance to gastric gas in patients with functional dyspepsia and may be beneficial for the treatment of gas‐related abdominal symptoms like bloating.

## 1 Introduction

Functional dyspepsia (FD) is one of the most common disorders of gut–brain interaction, characterized by alterations in gastric accommodation, gastric hypersensitivity, immune activation, and, in some cases, delayed gastric emptying [1, 2]. The main symptoms of dyspepsia encompass epigastric pain or burning sensation (typically in the epigastric pain syndrome subgroup [EPS]) and early satiety or postprandial fullness (in the postprandial distress syndrome subgroup [PDS]). In addition, up to 65% of patients with dyspepsia report gas‐related symptoms, such as abdominal bloating and abdominal distension, as bothersome complaints [3, 4].

The mechanisms underlying bloating have been previously addressed in several studies but remain incompletely understood [5, 6]. While many patients attribute bloating to an excess of intestinal gas, several studies have shown that patients with bloating have only minor increments in abdominal gas, which by themselves do not explain the magnitude of the symptoms raised by patients [7]. One of the mechanisms that has been largely involved in symptom development in functional dyspepsia is visceral hypersensitivity [8]. In this regard, recent studies with direct infusion of gas into the stomach have shown that patients with functional dyspepsia develop abdominal bloating in response to gas infusion, despite minor increments in gas retention; an effect that has been linked to hypersensitivity to gastric gas [9, 10]. More recently, changes in gut microbiome and duodenal immune activation have also been linked to symptom development in FD [2].

STW 5‐II (Iberogast N) is a herbal medicinal product, composed of six different herbal extracts (bitter candy tuft, caraway fruit, licorice root, peppermint leaf, lemon balm leaf, and chamomile flower). This formulation excludes three herbs (angelica root, milk thistle fruit, and greater celandine herb) present in STW 5. As STW 5, STW 5‐II has demonstrated a beneficial effect for management of functional dyspepsia in different controlled clinical trials [11]. These effects have been associated with the multitarget effects of this medicinal compound, which include modulation of visceral sensitivity [12, 13], gastric motor responses [14], and gut microbiome [15]. Therefore, the present study explored the proven effects of STW 5‐II on visceral sensitivity and gastroduodenal motility and its effect on tolerance to gastric gas, and specifically on gas‐induced abdominal bloating in patients with functional dyspepsia.

Our aim was to determine if previous treatment with STW 5‐II may improve gastric emptying of gas, as well as gas tolerance, in patients with functional dyspepsia, using a gas infusion methodology that has been previously developed and validated [9, 16].

## 2 Materials and Methods

### 2.1 Study Participants

Thirty‐two patients with FD (thirty‐one women and one man; age range 22–73 years) according to Rome IV criteria, and complaining of concomitant bloating, were included in the study. No patient met the criteria for irritable bowel syndrome (IBS). Patients were consecutively recruited from the out‐patient clinics of University Hospital Vall d'Hebrón in Barcelona, Spain. The diagnosis of FD was made using Rome questionnaires. According to Rome criteria, three patients had epigastric pain syndrome, 15 patients had postprandial distress syndrome, and 14 patients had overlapping symptoms. Inclusion criteria were being ≥ 18 years, Rome IV criteria for FD with active symptoms of bloating, and a negative upper endoscopy within 5 years. Exclusion criteria were known hypersensitivity to STW5‐II or one of the active substances or excipients, presence of organic gastrointestinal disease or major physical illness, any alteration in liver function tests, current alcohol or drug abuse and current or planned pregnancy or childbearing. All patients tested negative for *H. pylori* prior to enrollment. Table 1 shows the demographic characteristics of the patients. The protocol for the study was approved by the Spanish Agency of Medicines (Agencia Española de Medicamentos y Productos Sanitarios) and the Ethics Committee of the University Hospital Vall d'Hebrón. CONSORT reporting guidelines were used [17]. All patients provided written informed consent before participating in the study. All the authors had access to the study data and reviewed and approved the final manuscript. In the present manuscript the part two of the study (IBO‐2): FD patients treated with STW 5‐II, is presented.

**TABLE 1:** Demographic characteristics of the included patients.

	STW 5‐II group (*n* = 16)	PLACEBO group (*n* = 16)
Age (years), mean (SD)	45 (13)	49 (18)
Female, *n* (%)	15 (94%)	16 (100%)
Dyspepsia subtype		
EPS, *n* (%)	2 (12%)	1 (6%)
PDS, *n* (%)	7 (44%)	8 (50%)
Overlap, *n* (%)	7 (44%)	7 (44%)
Baseline PPI use, *n* (%)	6 (38%)	11 (69%)

*Note:* All data are *n* (%) unless otherwise specified.

Abbreviations: EPS, epigastric pain syndrome; PDS, postprandial distress syndrome; PPI, Proton Pump Inhibitors.

### 2.2 Gas Transit Test

#### 2.2.1 Gastric Gas Infusion

We used a polyvinyl tube assembly (3.2 mm outer diameter [OD]), that incorporated a gas infusion channel (1.6 mm inner diameter [ID]) with a distal opening positioned in the stomach. Gas was continuously infused at a rate of 100 mL/min for 15 min (1500 mL final volume) using a volumetric pump (GIP‐3000 Infusion Pump, Solfer Solucions SL, Barcelona, Spain). The gas mixture infused was comprised of 88% nitrogen, 6.5% carbon dioxide, and 5.5% oxygen, mimicking the partial pressures of venous blood gases to minimize diffusion across the intestine‐blood barrier.

#### 2.2.2 Nutrients Infusion

To emulate a standard meal, 315 kcal of a nutrient mixture (Fresubin YoDrink 1.5 kcal/mL; Fresenius Kabi, Barcelona, Spain) was continuously perfused at 14 mL/min for 15 min (210 mL final volume) into the stomach via a 1.2 mm ID perfusion channel incorporated into the nasogastric tube assembly, with the distal port located at the same level as the gas infusion site and connected to a volumetric pump (Alaris GH Guardrails Plus; BD Switzerland Sàrl. Eysins, Switzerland).

#### 2.2.3 Measurement of Rectal Gas Evacuation

Rectal gas evacuation was collected via an intrarectal Foley catheter (20 French, Bard, Barcelona, Spain) with the balloon inflated with 5 mL of water. The catheter was connected to a leak‐proof, low‐resistance collection line using a barostat, and the volume was continuously recorded on a personal computer for 90 min.

#### 2.2.4 Measurement of Gastric Belching

Gastric belching was continuously recorded using a pH‐impedance device (Z/pH Recorder, Diversatek, Highlands Ranch, CO). The pH‐impedance catheter was introduced transnasally into the stomach (drop of pH below 4), and then slowly withdrawn in 1‐cm steps to position the pH sensor 5 cm above the point at which the pH rises above 4 (esophageal pH). Belches were continuously recorded during the 90‐min study period.

### 2.3 Perception Measurements

Perception of the gastric gas loads was measured using a method that has been extensively used and previously validated in detail [16, 18, 19, 20, 21, 22, 23, 24]. Conscious perception was measured at 10‐min intervals using six graphic rating scales, each graded from zero (no perception) to six (painful sensation), specifically designed to score six types of abdominal sensations: (a) fullness/bloating, (b) hunger, (c) burning, (d) cramp/rumbling, (e) puncture/stinging sensation, and (f) other type of sensation (to be specified). The location of perceived sensations was marked on an abdominal diagram divided into nine regions corresponding to epigastrium, periumbilical area, hypogastrium, both hypochondria, flanks, and iliac fossae. Participants were instructed to report the sensations perceived over the preceding 10‐min period using the scales. Belches were self‐recorded by the patient by pressing a button of the pH‐impedance recording device each time a belch was identified. Based on previous observations, we anticipated that gas should leave the stomach very quickly to lower intestinal segments and, consequently, we assessed not only epigastric typical dyspeptic symptoms, but also symptoms referred to other parts of the abdomen [16, 18, 25, 26].

### 2.4 Measurements of Abdominal Distension

Once the subjects were positioned in bed, a non‐stretch metric tape measure was adjusted around the abdomen above the umbilicus using two elastic bands. While the subjects were breathing in a relaxed manner, girth measurements were taken at 10‐min intervals as the average of inspiratory and expiratory determinations over three consecutive respiratory excursions [27].

### 2.5 Procedure

After a 2‐week period on treatment with STW 5‐II or placebo (see Section 2.6), on the day of the study, patients came to the Motility Unit after an 8‐h fast. Participants were instructed to follow a low‐flatulogenic diet during the 2 days prior to the study day, avoiding fruit, vegetables, legumes, and whole grain products (bread, cookies). The night before the study, patients were instructed to have a light dinner consisting of meat, fish, eggs, rice, pasta, and/or white bread, while avoiding dairy products, salad, fruits, and alcoholic beverages. If the patient had not passed stools within 8 h before the study, bowel evacuation was induced by administration of a rectal enema with 250 mL of saline (Fisioenema, Casen Recordati, Utebo [Zaragoza], Spain). The use of medications that may interfere with gastrointestinal motility (bulking agents, laxatives, linaclotide, prokinetics, antidiarrheal, or opioids) was discontinued 48 h before the gas infusion test. The morning dose of placebo or STW 5‐II, as scheduled, was administered as usual before the gas transit test.

First, with the patient in a lateral supine position, the rectal tube was introduced and connected to the barostat. Then, the patient was asked to sit at a 45° angle horizontally, and the nasogastric gas infusion catheter was introduced into the stomach transnasally and fixed at 45 cm from the nose. Finally, the pH‐impedance catheter was introduced also transnasally and positioned as previously described. The exact point of the esophago‐gastric junction was established as the point of acidification of the pH (pH < 4), and the nasogastric infusion tube was repositioned if necessary to ensure that the infusion port was located 5 cm below this point. Both catheters were fixed in place by taping them to the nose. Gastric gas infusion and nutrient perfusion were started with the participant lying in the supine position at a 30° angle horizontally and maintained for 15 min. From the start of gas and nutrient infusion, belching and rectal gas evacuation were continuously recorded for 90 min, while perception and abdominal distension were measured at 10‐min intervals over the same period.

### 2.6 Experimental Design

A parallel, randomized, double‐blinded, placebo‐controlled study was designed to determine the effect of STW 5‐II on gastric gas tolerance. Eligible patients were randomized on a 1:1 basis to receive either STW 5‐II or identical‐appearing placebo, according to centralized automated randomization. The trial was double‐blind; neither the patients, research team, nor treating staff knew the treatment allocation. This was achieved through identical packaging, labeling, and organoleptic properties (liquid presentation, dark brown color, taste, and smell) of both the STW 5‐II and matched placebo bottles. Each bottle of STW 5‐II or placebo was identified by a kit code. Randomization lists containing kit allocation were generated by the supply company providing the kits (Steigerwald Arzneimittelwerk GmbH, Darmstadt, Germany). Code‐break envelopes were also provided by the supply company, and access to the envelopes was restricted to the designated safety team.

In each participant, the gas challenge test was performed after a 2‐week intervention period with the patient on either placebo or STW 5‐II (20 drops t.i.d.). The gas challenge test was conducted with both the investigators and patients blinded to the assigned intervention. Adverse events were assessed after the intervention period before the start of the corresponding gas infusion test.

### 2.7 Data Analysis

In each subject, rectal gas evacuation was calculated as the sum of the gas collected from the rectal cannula during the study period. The volume of gas retained within the gut was calculated as the difference between the volume of gas infused and the volume of gas recovered. The number of belches was also calculated in each patient, following previously described criteria [16, 28, 29]. Perception of abdominal sensations was assessed using the score rated on the scales at each time interval. When more than one sensation was reported at the same time point, the highest score (i.e., the most severe sensation regardless of type) was used for comparisons.

For each subject, the number of times each abdominal sensation was reported across repeated measurements during the study was counted to calculate the frequency (as percent distribution) of each specific sensation. In the anatomical questionnaire, the percentage of sensations referred to each abdominal region, as well as the percentage reported in more than one region, was calculated. This methodology has been widely used and validated in previous studies [9, 16, 28].

Changes in abdominal girth during the study were referenced to the baseline girth measurement obtained before gas infusion was initiated.

### 2.8 Statistical Analysis

Based on data from similar studies [9, 10], we considered that patients with functional dyspepsia should retain about 360 mL of gas at the end of gas/nutrient infusion, and that pretreatment with STW5‐II should normalize the volume of gas retained to a level similar to that observed in healthy subjects and patients with functional dyspepsia without nutrient infusion (about 30 mL of gas retention at the end of the infusion period) with great interindividual variability (±300 mL). Considering this and a 10% dropout, 16 patients per group should complete the study to discriminate differences in gas retention with a power of 80% and an alpha error of 5%.

In each treatment group (placebo or STW‐II), mean values (±standard error [SE]) of the parameters measured at 10‐min intervals were calculated. The Kolmogorov–Smirnov test was used to assess the normality of data distribution. Comparisons of repeated, parametric, normally distributed data were performed using the ANOVA test for repeated measures. Post hoc analyses were performed by the Student's *t*‐test for unpaired data. Non‐parametric data were compared using the Mann–Whitney *U* test. Intra‐group comparisons of data before versus the end of gas infusion were performed using the paired Student's *t*‐test or the Wilcoxon signed‐rank test, as appropriate. The frequency and location of symptoms were compared using the Mann–Whitney *U* test. The linear correlation coefficient was used to compare registered and recorded belches.

## 3 Results

### 3.1 General Aspects

**FIGURE 1:**
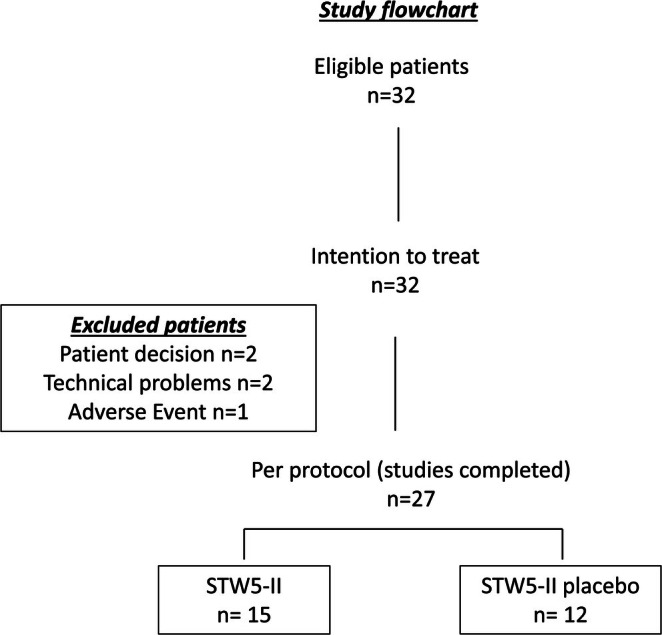
Study flowchart.

Twenty‐seven subjects completed the study protocol uneventfully. The reasons for withdrawal of the remaining five patients were patient decision in three cases and technical issues with the gas‐recovery system in two cases (Figure 1).

A single adverse event was reported during the study: a mild urinary tract infection occurred during treatment with placebo, which resolved spontaneously. However, the patient chose to discontinue her participation in the study. No adverse events were reported during STW 5‐II intake.

### 3.2 Effect of STW 5‐II on Gas Transit During the Gas Challenge Test

**FIGURE 2:**
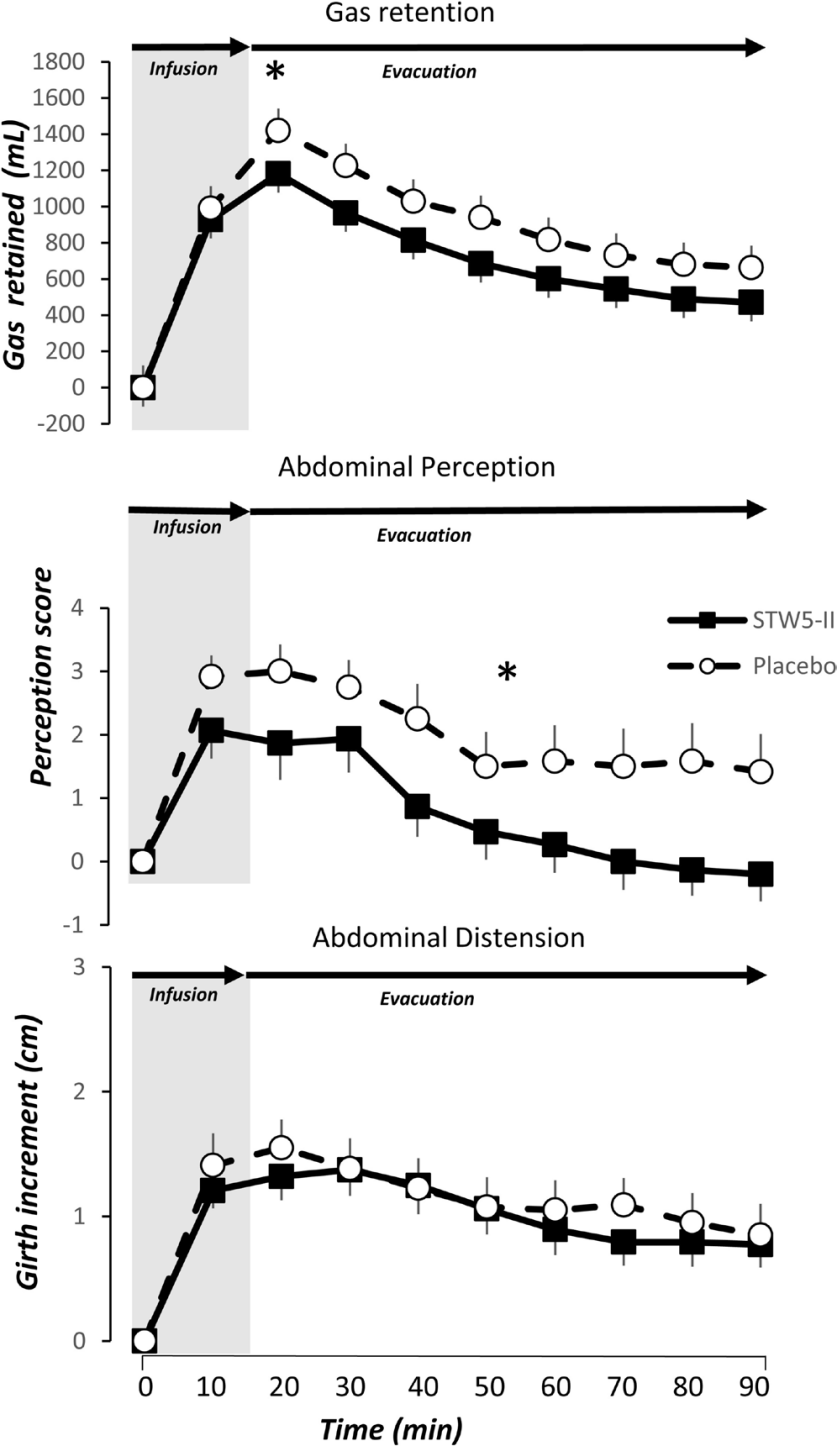
Effect of gastric gas and nutrient infusion on gas retention, abdominal perception, and abdominal distension. Studies performed on STW5‐II showed a faster evacuation of gas and a lower perception of abdominal symptoms. There were no differences in abdominal girth. *p < 0.05 vs. placebo.

Gastric gas infusion (15 min period) was followed by a progressive start of gas evacuation from the rectum during the following 90 min. At the beginning of the study, the rate of gas evacuation was significantly lower than the infusion rate, leading to transient gas retention in the intestine, which was progressively reduced during the evacuation period (Figure 2). However, rectal gas evacuation was significantly accelerated when patients were on STW 5‐II compared to placebo. Hence, 20 min after the start of infusion—when the 1500 mL of gas had been infused into the stomach—only 80 ± 39 mL of gas had been expelled in the placebo group, whereas 319 ± 81 mL of gas had been expelled in the STW 5‐II group (*p* = 0.015). Thereafter, both groups continued to expel gas at similar rates, with no significant differences in the total amount of gas retained at the end of the study between the treatment groups (470 ± 160 and 662 ± 179, on STW 5‐II and placebo, respectively, *p* = 0.431).

### 3.3 Effect of STW 5‐II on Belches During the Gas Challenge Test

During the first 20 min of gas infusion, after 1500 mL of gas has been infused into the stomach over 15 min, 1.07 ± 0.45 and 0.81 ± 0.4 gastric belches were registered on placebo and STW 5‐II, respectively (*p* = 0.67). At the end of the 90‐min study period, only 1.61 ± 0.67 and 2.18 ± 1.09 gastric belches were registered (placebo and STW 5‐II, respectively; *p* = 0.33). Gastric belching was exceptional throughout the study period, indicating that rectal gas evacuation was the main route of gas elimination.

Supra‐gastric belching (SGB) was also analyzed. At the end of the study, 0.84 ± 0.60 and 1.63 ± 0.72 SGB episodes were registered in STW 5‐II and placebo studies, respectively (*p* = 0.20). Supra‐gastric belches were also exceptional throughout the entire study period.

### 3.4 Effect of STW 5‐II on Abdominal Symptoms During the Challenge Test

Infusion of gas into the stomach was associated with a rapid and significant increase in the perception of abdominal symptoms (mainly bloating), reaching its peak at 20 min after the start of infusion, when the full 1500 mL of gas had been completely infused (Figure 2). However, the acceleration of gas transit and evacuation observed in patients treated with STW 5‐II was associated with a smaller increase in perception compared to those receiving placebo, and with a faster decline and return back to baseline levels of the intensity of abdominal symptoms (Figure 2).

Considering the entire study period, significantly lower perception scores were repeatedly reported by patients treated with STW 5‐II compared to those receiving placebo (*p* = 0.035, repeated measures ANOVA test).

**FIGURE 3:**
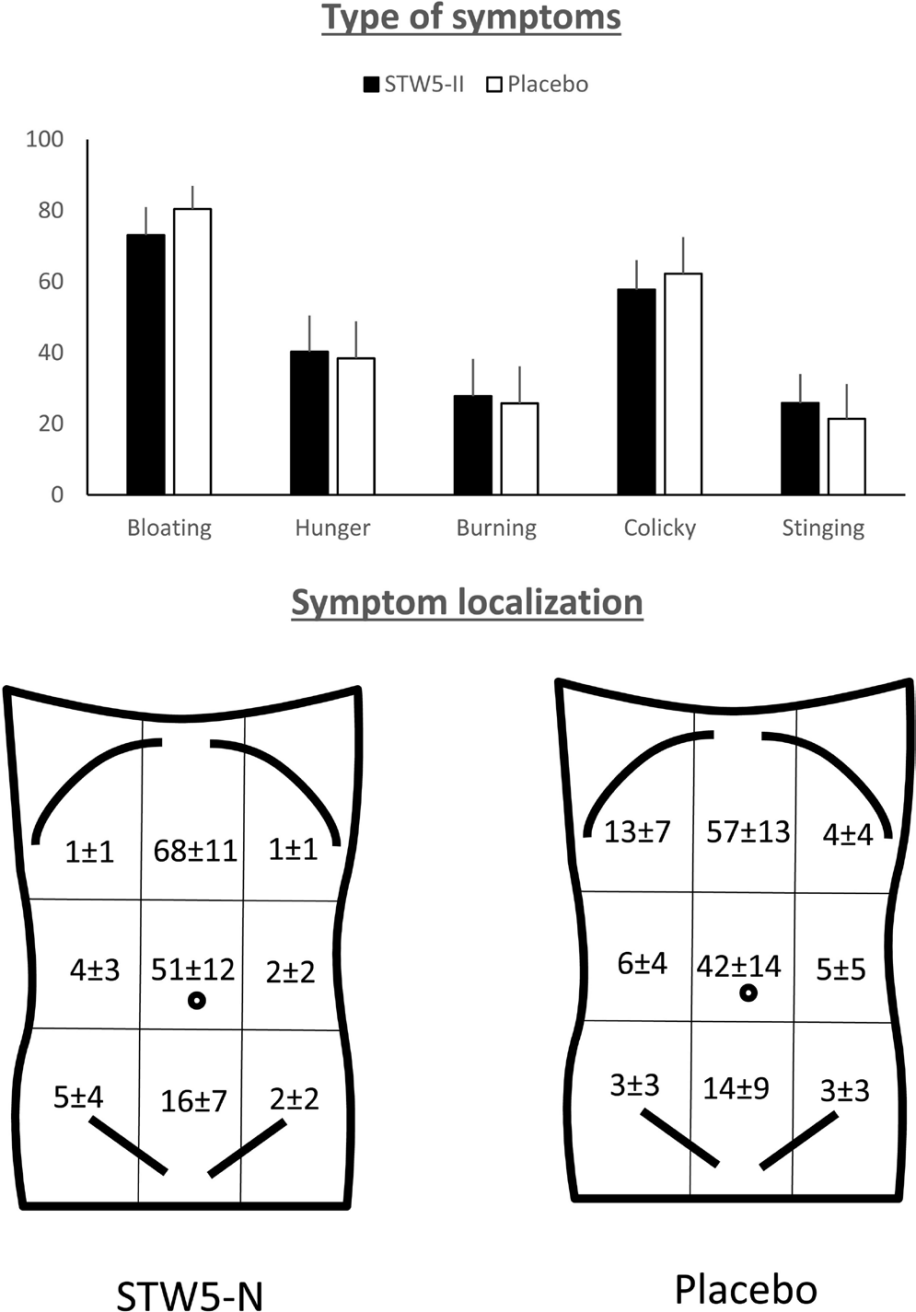
Type and localization of abdominal symptoms during gastric gas infusion. The most common reported symptoms during the gas challenge test, either STW5‐II or placebo, were bloating and colicky sensation. Most of the time the abdominal perception was located in the abdominal midline, mainly on the epigastrium and periumbilical area.

The most frequently reported type of abdominal perception was bloating/fullness sensation, accounting for over 70% of times, either alone or in combination with hunger, burning, cramp/colicky, or stinging sensation. There were no significant differences regarding whether the infusion was performed on STW 5‐II or placebo (*p* = 0.383) (Figure 3).

Most sensations were referred by patients to the abdominal midline, predominantly in the epigastric and periumbilical areas (Figure 3). There were no differences in the extent of the referred area between groups: 30% ± 9% of sensations were referred to more than one abdominal area in the STW 5‐II group, compared to 38% ± 7% in the placebo group (*p* = 0.235).

### 3.5 Effect of STW 5‐II on Objective Abdominal Distension During the Gas Challenge Test

Like abdominal perception, gastric gas infusion was associated with a rapid increase in abdominal girth, reaching its peak at 20 min after the start of infusion, when the full 1500 mL of gas had been completely infused (Figure 2). The increase in abdominal girth at 20 min was comparable between the STW 5‐II and placebo groups (girth increase: 13 ± 2 mm vs. 16 ± 2 mm, respectively, *p* = 0.224). Thereafter, abdominal distension decreased slightly but remained significantly greater than baseline in both groups: girth increase of 8 ± 3 mm in the STW 5‐II group (*p* < 0.001 vs. baseline) and 9 ± 2 mm in the placebo group (*p* = 0.003 vs. baseline).

## 4 Discussion

In the present study, the effect of STW 5‐II on transit and tolerance to gastric gas in patients with FD and concomitant bloating was evaluated in a double‐blind, placebo‐controlled clinical trial. These results show that STW 5‐II accelerates gastric gas evacuation in dyspeptic patients, reducing early gas retention following gastric loading. Furthermore, gastric gas produces a rise in abdominal symptoms, more frequently referred to as bloating/fullness, that are attenuated by STW 5‐II compared to placebo.

Previous studies in healthy volunteers using the same methodology of direct gas infusion into the stomach [16] have shown that under normal physiological conditions, gas reaching the stomach is promptly emptied into distal intestinal segments and finally expelled via the rectum, producing minimal abdominal symptoms. In contrast, dyspeptic patients exhibit a transient delay in gas evacuation during the initial minutes following gastric gas infusion [9], and subsequent studies have shown that this delay is exacerbated when gas infusion is combined with nutrient intake, mimicking the postprandial state.

Additionally, patients with FD develop significantly more pronounced abdominal symptoms during gas infusion into the stomach, an effect associated with visceral hypersensitivity that characterizes disorders of the gut–brain axis interaction. Notably, prior studies also showed that the belching reflex is only triggered by massive gastric gas loading at high infusion rates (e.g., 400 mL/min) in healthy individuals. At physiological infusion rates, comparable to those during meals, belching increases only in patients with a belching disorder, but not in healthy controls or FD patients.

Our findings confirmed most of these previous observations. After 2 weeks on placebo, dyspeptic patients showed a delayed onset of rectal gas expulsion, preceded by transient intraluminal accumulation, a rise in abdominal perception, and measurable abdominal distension. In contrast, after 2 weeks of pre‐treatment with STW 5‐II, rectal evacuation of gas was significantly accelerated, and the increase in abdominal symptoms was consistently lower throughout the study period. Likewise, despite the fact that gas infusion induced a rapid onset of moderate‐to‐severe epigastric symptoms, no increase in belching to decompress the stomach was observed in either treatment arm, confirming that at physiological infusion rates, gastric gas is predominantly transferred to the distal intestine and eliminated rectally.

The mechanisms by which STW 5‐II accelerates rectal gas evacuation and improves tolerance to gastric gas likely result from the combined effects of its multiple actions along the gastrointestinal tract, as demonstrated in previous studies. Human studies have shown that STW 5‐II induces relaxation of the gastric fundus and increases antral contractility [14]. Additional in vitro studies have reported that several components of STW 5‐II decrease muscular activity in the proximal stomach while enhancing contractile activity in the antrum [30].

In addition to its effects on gastrointestinal motility, several studies have demonstrated that STW 5‐II reduces visceral sensitivity. In animal models, STW 5‐II was shown to reduce intestinal afferent nerve discharge in response to intravenous bradykinin, a key mediator of nociception [12]. Thus, the combination of improved gastroduodenal motility and reduced visceral sensitivity may synergistically contribute to enhanced tolerance to gastric gas observed in patients treated with STW 5‐II. However, the specific role of each one of these putative mechanisms in the effects of STW 5‐II on gastric gas transit and tolerance cannot be elucidated from our study, and future research using a methodological approach aimed to discriminate mechanistic effects in response to gastric gas are required.

By contrast to symptom perception, no differences in objective abdominal distension were observed between patients treated with STW 5‐II and placebo. Previous studies have shown that, in patients with bloating and disorders of gut–brain interaction, abdominal distension is produced by altered abdominophrenic responses to small increments in abdominal contents, so‐called abdominophrenic dyssynergia [31]. Our results suggest that STW 5‐II exerts its main effects on visceral sensitivity, but new studies monitoring the diaphragmatic and abdominal muscle responses are needed to evaluate its potential effects on abdominophrenic responses to gastric gas loads.

In the present study, we used a newer variant of the original STW 5 formulation, which contains only 6 of the 9 original herbal extracts. The revised formulation includes higher concentrations of licorice root and chamomile flower, as well as an extract of the full *Iberis Amara* plant. For decades, the original STW 5—comprising nine herbs—has been widely used as an effective herbal remedy for FD and DGBI, with multiple iterations having undergone clinical testing. STW 5‐II represents a simplified version of this formulation, yet it has demonstrated significant clinical efficacy in treating FD as well [14, 32].

An “in vitro” study by Simmen et al. [33] demonstrated that STW 5 binds to various receptors involved in pain transmission, including serotonin, muscarinic, and opioid receptors. In this study, the authors analyzed separately the effects of the different herbal components of STW 5 and found that the fresh plant extract of *Iberis Amara* , one of the main components of STW 5‐II, selectively inhibited M3 receptor binding, while chamomile flower and licorice root extracts bound to 5HT_4_ and 5HT_3_ receptors, respectively.

Beyond receptor binding, components of STW 5‐II have also shown antispasmodic effects comparable to those of papaverine, which may contribute to the reduction in symptom perception reported by patients during gastric gas infusion [34, 35]. These experimental findings support the rationale for the observed reduction in abdominal pain in irritable bowel syndrome (IBS) patients treated with STW 5 [36], as well as the decreased bloating induced by colonic gas infusion in patients with IBS [37].

In the present study, we used a gas and nutrients infusion methodology that has been previously validated in studies involving healthy individuals and patients with functional dyspepsia [9, 16, 37]. This methodology consists of a gas challenge test with direct infusion of gas and nutrients into the stomach. It offers the advantage of precisely controlling the amount of gas and nutrients reaching the stomach, allowing for the performance of comparative studies under controlled conditions. However, a limitation of the study is that it was performed in a non‐physiological situation, under laboratory conditions. Moreover, to properly challenge the stomach, the amount of gas infused (1500 mL) corresponds to the upper limit of what is typically ingested during a standard meal. This data is calculated from the results of previous studies showing that in a standard meal as much as 40 swallows containing gas can occur [28], and that each swallow may contain up to 32 mL of gas [38]. In addition to swallowed gas, food and beverages containing different amounts of gas may also increase the volume of gas entering the stomach during a meal. However, no studies have assessed gas swallowing during meals in dyspeptic patients.

Another limitation of the study includes the small sample size, which had an overrepresentation of women with the PDS and mixed subtypes of functional dyspepsia. Consequently, the generalizability of the findings to other FD subtypes, such as EPS, or to male populations, remains limited.

In conclusion, this study demonstrated that the herbal medicinal product STW 5‐II—comprising six plant extracts with complementary actions on gastrointestinal motility and visceral sensitivity—improves tolerance to gastric gas in patients with functional dyspepsia and concomitant bloating. These results suggest that this formulation may be beneficial in the treatment of dyspeptic patients with associated bloating symptoms.

## Author Contributions

A.A.: acquisition of data; analysis and interpretation of data; drafting the manuscript, statistical analysis. L.A., C.B., J.S., B.L., and C.M.: patient selection and inclusion. J.S.: study concept and design, analysis and interpretation of data; critical revision of the manuscript for important intellectual content.

## Conflicts of Interest

J.S.: research grants from Bayer and Salvat laboratories; and consultant/speaker with Menarini, Casen Recordati, Reckitt Benckiser, and Norgine. The other authors declare no conflicts of interest.

## Data Availability

The authors have nothing to report.
